# Supplementary material for: Characterization of Vegetative Bacillus cereus and Bacillus subtilis Strains Isolated from Processed Cheese Products in an Italian Dairy Plant
Source: Foods. 2021 Nov 21;10(11):2876. doi: 10.3390/foods10112876 (PMC8622485; doi:10.3390/foods10112876)
Supplement: Supplementary file 1 [file foods-10-02876-s001.zip › foods-1463060-supplementary.pdf]

Table S1: Isolated *Bacillus* strains included in the study

ID strain	Organism	Collection period
BC_2	*Bacillus cereus*	Summer
BC_3	*Bacillus cereus*	Summer
BC_5	*Bacillus cereus*	Summer
BC_14	*Bacillus cereus*	Summer
BC_19	*Bacillus cereus*	Summer
BC_22	*Bacillus cereus*	Summer
BC_24	*Bacillus cereus*	Summer
BC_26	*Bacillus cereus*	Autumn
BC_29	*Bacillus cereus*	Autumn
BC_35	*Bacillus cereus*	Autumn
BC_36	*Bacillus cereus*	Autumn
BC_38	*Bacillus cereus*	Autumn
BC_40	*Bacillus cereus*	Autumn
BC_44	*Bacillus cereus*	Autumn
BS_8	*Bacillus subtilis*	Summer
BS_15	*Bacillus subtilis*	Summer
BS_17	*Bacillus subtilis*	Summer
BS_18	*Bacillus subtilis*	Summer
BS_20	*Bacillus subtilis*	Summer
BS_23	*Bacillus subtilis*	Summer
BS_25	*Bacillus subtilis*	Summer
BS_28	*Bacillus subtilis*	Summer
BS_31	*Bacillus subtilis*	Summer
BS_34	*Bacillus subtilis*	Summer
BS_41	*Bacillus subtilis*	Autumn
BS_42	*Bacillus subtilis*	Autumn
BS_48	*Bacillus subtilis*	Autumn
BS_49	*Bacillus subtilis*	Autumn
BS_50	*Bacillus subtilis*	Autumn
BS_51	*Bacillus subtilis*	Autumn
BS_52	*Bacillus subtilis*	Autumn
BS_53	*Bacillus subtilis*	Autumn
BS_54	*Bacillus subtilis*	Autumn
BS_55	*Bacillus subtilis*	Autumn
BS_57	*Bacillus subtilis*	Autumn
